# Cashing in: cost-benefit analysis framework for digital hospitals

**DOI:** 10.1186/s12913-024-11132-7

**Published:** 2024-05-31

**Authors:** Kim-Huong Nguyen, Tracy Comans, Thi Thao Nguyen, Digby Simpson, Leanna Woods, Chad Wright, Damian Green, Keith McNeil, Clair Sullivan

**Affiliations:** 1 Faculty of Medicine, The University of Queensland, Brisbane, Australia; 2 Global Brain Health Institute, Trinity College Dublin, Dublin, Ireland; 3 School of the Environment, The University of Queensland, Brisbane, Australia; 4 Queensland Digital Health Centre, Faculty of Medicine, The University of Queensland, Brisbane, Australia; 5 eHealth Queensland, Brisbane, Australia; 6 Queensland Department of Health, Brisbane, Australia; 7 Metro North Hospital and Health Service, Herston, Australia; 8 Brain and Mind Centre, Faculty of Medicine and Health, University of Sydney, Sydney, Australia; 9 National Ageing Research Institute, Parkville, Victoria, Australia

**Keywords:** Digital hospitals, Electronic medical records, Economic evaluation, Cost benefit analysis, Total economic value

## Abstract

**Background:**

For many countries, especially those outside the USA without incentive payments, implementing and maintaining electronic medical records (EMR) is expensive and can be controversial given the large amounts of investment. Evaluating the value of EMR implementation is necessary to understand whether or not, such investment, especially when it comes from the public source, is an efficient allocation of healthcare resources. Nonetheless, most countries have struggled to measure the return on EMR investment due to the lack of appropriate evaluation frameworks.

**Methods:**

This paper outlines the development of an evidence-based digital health cost-benefit analysis (eHealth-CBA) framework to calculate the total economic value of the EMR implementation over time. A net positive benefit indicates such investment represents improved efficiency, and a net negative is considered a wasteful use of public resources.

**Results:**

We developed a three-stage process that takes into account the complexity of the healthcare system and its stakeholders, the investment appraisal and evaluation practice, and the existing knowledge of EMR implementation. The three stages include (1) literature review, (2) stakeholder consultation, and (3) CBA framework development. The framework maps the impacts of the EMR to the quadruple aim of healthcare and clearly creates a method for value assessment.

**Conclusions:**

The proposed framework is the first step toward developing a comprehensive evaluation framework for EMRs to inform health decision-makers about the economic value of digital investments rather than just the financial value.

**Supplementary Information:**

The online version contains supplementary material available at 10.1186/s12913-024-11132-7.

## Introduction

Health services are increasingly required to demonstrate that their investments in digital transformation result in benefits or value [1]. Digital hospitals, representing substantial investments in infrastructure, technology, and human resources, are at the forefront of this evolution. However, the rapid rate of digital transformation in healthcare surpasses the rate at which innovations can be monitored and evaluated [2], complicating efforts to demonstrate the value of digital health [3]. Concerns regarding returns on investment, competing priorities (particularly diverting funds from routine healthcare) and the perception of high risk [4] further impede progress in understanding the value of digital transformation [5].

Despite the increasing frequency of economic evaluations in the literature, the evidence on how best to evaluate large-scale digital health investments such as electronic medical records (EMRs) is incomplete. A comprehensive scoping review by Nguyen et al. (2021) found significant heterogeneity of evaluation settings and measurement methods in assessing the impacts of EMR implementation [6]. These include (a) the large variance in implementation conditions and scope of EMR, (b) the wide range of analysis units, measures and indicators at both patient and health system levels, and (c) a variety of analytical methods which range from short term financial analyses to longitudinal econometrics [6].

This heterogeneity reflects the early stage of measuring and valuing the impacts of digital health transformation. For instance, except a few studies [7–9], existing evidence is limited to only partial economic evaluation or impact assessments. The “non-cashable” nature of many quality-improvement measures (e.g., patient experience) is a new concept and poorly understood in financial-based evaluation models. Traditional financial models are also limited to a short term and have inadequate timeframes post-EMR implementation. The immediate implementation period (5 years or less) [10–12], instead of medium (7–10 years) or long-term periods (10 + years), have traditionally been considered. More recently, it is understood that it often takes at least 3 years post-implementation to observe the emergence of some benefits of a transformational digital health implementation [13, 14]. Standardisation of measures and valuation methods are particularly important to address this uncertainty within the literature.

In addition to the methodological challenges, the health system goals (e.g., equity, efficiency, accessibility, responsiveness) have changed steadily to meet the fast-changing healthcare landscape. The quadruple aim has recently been recognised as an aspiration for healthcare systems internationally. The aims include (1) to improve population health, (2) to enhance patient experience, (3) to reduce cost per patient, and (4) to improve the work-life balance of the healthcare workforce [15]. Due to its relative newness, this has not yet been reflected in digital health evaluation. For instance, the digital health evaluations, driven by the traditional business-case financial-based models, have focused mostly on “the cost per patient” aspect, and occasionally, patient outcomes. Consumer experience, and in particular, the experience of the healthcare workforce, is neglected. As a result, health systems that view the implementation and maintenance of an EMR as “inevitable” struggle to justify the investment and operation costs to funders outside of incentivised EMR roll-out schemes [16].

Investment in digital health is a long-term investment in infrastructure and workforce, which is complex to implement, appraise and evaluate. It is, therefore, essential to have a comprehensive account of costs and benefits in both financial and economic values, a transparent breakdown of the cost-benefit distribution, and over different time horizons (from short to long-term). The sole focus on short-term financial return and the delivery of the benefits to the funder alone (as opposed to users and consumers) have been slowly replaced by a broadened view of what constitutes “value” in healthcare investment [17]. This maturity constitutes a shift from traditional financial assessments to economic assessments that include intangible impacts such as consumer satisfaction, workforce wellbeing, and family spillover impact. Applying full economic assessments to large digital health projects is challenging and labour-intensive as there is little evidence or framework to guide investigators and funders.

These complex investment projects involve multiple stakeholders whose costs and benefits might not be equally distributed. For instance, in universal healthcare systems like Australia and Canada, the main beneficiaries of investment in patient safety are patients who enjoy improved health outcomes but do not have to incur additional payments (or any payments at all). The cost bearer, in this case, is the hospital and/or the state that funds the digital health system. Another stakeholder group that might experience negative impacts of an EMR implementation in the short term is healthcare workers who undertake discretionary efforts to revise their workflow to fit the new system. This can lead to burn-out and/or a temporary reduction in job satisfaction and adverse clinical events during the implementation stage. Without breaking down the benefit and cost streams by individual stakeholder groups, it is not possible to understand factors contributing to a successful/failed reform initiative and opportunities to improve the project design to reduce the negative effects on a particular group of stakeholders, and/or enhance the economic benefits of the overall project.

Responding to the growing need for a comprehensive framework to understand the economic value of EMRs and other digital health initiatives, we develop a systematic Cost-Benefit Analysis (CBA) approach to evaluate the resource allocation in digital health investment. This framework aims to ensure that scarce resources are directed towards initiatives that yield the highest returns regarding patient care, operational efficiency, and long-term sustainability. CBA framework can provide decision-makers with the necessary insights to assess the financial viability of digital initiatives, including considerations such as cost savings from capital and operational expenditure, waste reduction and revenue generation. By conducting comprehensive cost-benefit analyses, healthcare organizations can make informed decisions that optimize financial performance while maximizing the value delivered to patients and stakeholders. Through CBA, stakeholders can assess digital interventions’ direct and indirect impacts on patient outcomes, such as reduced hospital readmissions, improved medication adherence, and enhanced patient satisfaction. CBA can enable a nuanced examination of how digital hospitals can address healthcare disparities and promote health equity. By identifying and quantifying the differential impacts of digital interventions on underserved populations, policymakers can prioritize initiatives that narrow gaps in access to care, improve health outcomes among vulnerable populations, and foster inclusive healthcare delivery models. Additionally, CBA can enable stakeholders to gauge the impact of digital interventions on healthcare workforce satisfaction, workload distribution, and professional development, thereby fostering a supportive and resilient healthcare workforce capable of delivering high-quality care amidst evolving challenges.

In this paper, we will report on developing an eHealth-CBA framework. The framework has been applied to appraise a digital hospital investment in Australia (completed in 2021) and is currently guiding further data collection to validate its original findings (appraisal).

## Method

**Fig. 1:**
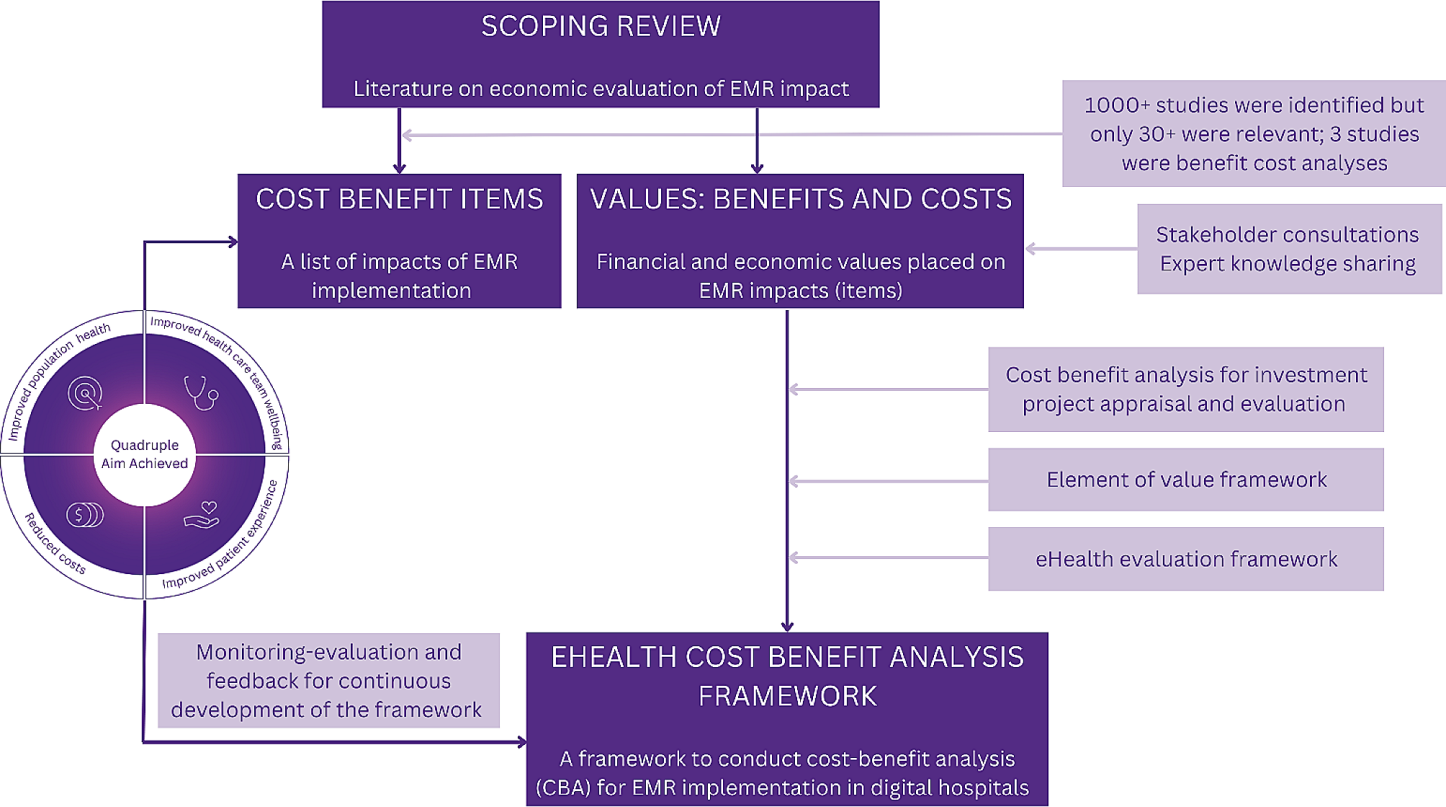
Summary of the three-stage process to develop the eHealth CBA. *Abbreviation* EMR: electronic medical records; CBA: Cost-benefit analysis

The digital health cost-benefit analysis (eHealth-CBA) framework was developed in a three-stage process (see Fig. 1). While there is a natural sequence from stages 1 to 3, the process also follows a continuous feedback loop between stages to account for recent advancements in eHealth and economic evaluation methods, the local knowledge and experience in reference to the quadruple aims of healthcare, and data availability and data collection timeframe. This study was granted ethical approval by the Human Research Ethics Committee (HREC) [project HREC/2020/QRBW/69,963 and project HREC/2020/QRBW/66,895].

The research started with the premise that there are many different ways to appraise and evaluate a digital health investment project, of which the social cost-benefit analysis is a potential approach due to its comprehensive process that accounts for multiple stakeholders and the project’s impacts on them.

### Stage 1 – literature review

We undertook a comprehensive scoping review to assess the current methodologies and proposed costs and benefits of EMR implementation published since 2000 [6]. The review results were reported in a separate publication (Nguyen et al. 2021) and reflect three core themes. These themes were deemed important to consider when developing a digital health cost-benefit analysis framework, including (1) the maturity of EMR and digital hospitals, (2) economies of scope and scale, and (3) the alignment of EMR with health system goals. These informed the evaluation timeframe and identification of benefit and cost items for the CBA.

The literature search also pointed us toward approaches that could contribute to the framework development. Two approaches, the “elements of value” [19] and the digital health benefit evaluation [20], were deemed most complementary to the CBA approach. These two approaches were summarized in the Appendix.

### Stage 2 – stakeholder consultation

A systematic consultation process was adopted with relevant stakeholders belonging to the Referent Group. For the digital hospital CBA analysis, the Reference Groups include patients, staff, the hospital as an entity, and the broader health district the hospital serves. The consultation includes a series of workshops to discuss the initial findings from the literature review (Stage 1), a series of follow-up consultations to capture the perspectives of different health authorities, digital health subject matter experts and hospital and administrative staff, and the result consultations to confirm the feasibility and acceptability of the framework (after Stage 3) amongst stakeholders. During the framework development (stage 3), multiple stakeholders were invited to validate the framework (especially the benefit and cost items and how they should be measured) as it was developed. Content analysis of the minutes was conducted by the project members following each consultation to aggregate findings to incorporate into improvements to the eHealth-CBA framework (Further details can be found in the Appendix).

### Stage 3 – CBA framework development

This stage aims to develop a CBA framework to evaluate EMR implementation in hospitals, accounting for the quadruple aim of healthcare, the digital health transformation context of Australia, the existing literature on healthcare economics (evaluation methods, and “elements of value” framework), valuation of costs and benefits associated with eHealth implementation, and the local practice and experience by the stakeholder (hospital workforce, healthcare/hospital decision-makers and patients/consumers). The framework was improved through stakeholder engagement and feedback with a workshop and follow-up consultations (Stage 2). This has resulted in the first comprehensive economic evaluation framework developed for EMR implementation to inform decision-makers of the value of their digital investments.

**Fig. 2:**
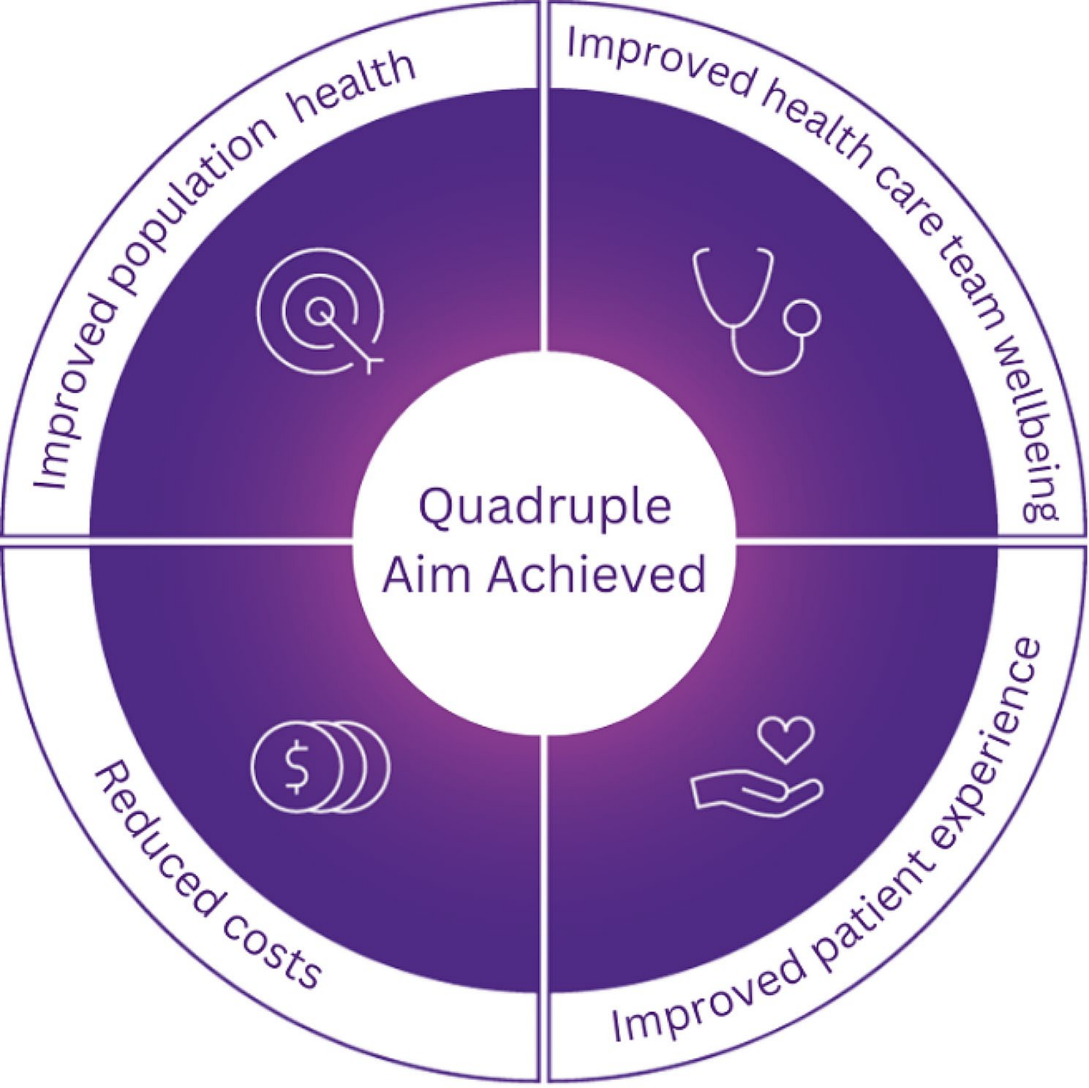
Quadruple aim of healthcare delivery

The key components of the framework consist of:

1. Cost-benefit analysis: Cost-benefit analysis (CBA) is a process of identifying, measuring and comparing the benefits and costs of an investment program or project, typically based on economic data derived from the estimated *opportunity costs* of an alternative use for the same resources [18]. More recent developments in CBA have recommended including non-market criteria (or non-financial valuation) to gauge a broader and more appropriate range of positive and negative externalities [19]. A positive net-benefit indicates that the project is an efficient investment of resources, compared to its alternatives, from an economic point of view. While the net-benefit resembles the “profit” concept in financial analysis, the key difference here is that the former contains non-market values of benefits and costs (opportunity costs) while the latter only covers market prices of the (investment) outputs and resources used. All outputs or inputs that do not have an “explicit” market (e.g., patient quality of life, workforce satisfaction, safety measures, etc.) are not counted in a financial analysis. When performing a CBA, it is crucial to identify all the benefits and costs to include in the four parts of the analysis (Market, Private, Efficiency and Reference Group) [18]. Some items might be relevant for one type of analysis only. Some items might have the same or different values in different types of analysis. This section will discuss all items that should be quantified and included in the CBA.

2. The quadruple aim of health care: the four aims focus on healthcare service delivery, including (1) to improve population health, (2) to enhance the patient experience, (3) to reduce cost per patient, and (4) to improve the work-life balance of healthcare workforce (Fig. 2). It was developed by Bodenheimer and Sinsky (2014) [15] and has been instrumental in discussing the success and failure of healthcare system performance. The quadruple aim of healthcare can be expanded further as needs arise, following the evolution of the modern healthcare system.

3. The elements of value framework: the Professional Society for Health Economics and Outcomes Research (ISPOR) special task force report by Lakdawalla et al. (2018) [19] discusses a series of elements that warrant consideration in value assessments of health and medical technologies. They aimed to broaden the view of what constitutes value in health care and urged for new research of the additional elements.

4. The eHealth evaluation framework: developed by Lau and Kuziemsky (2016) [20], presented elements of successful information systems in different settings [21, 22], systematic reviews on the determinants of success in inpatient clinical information systems [23], and synthesis of results from health information system evaluations [24]. It stops at naming the benefit items. The economic values of benefit items were not presented or discussed, making it impossible to use them for an economic evaluation.

Lastly, a follow-up valuation (i.e. validation) was proposed and scheduled to happen within the first five-year of the digital hospital implementation. This reflects the opportunities to collect data following the EMR implementation and to validate the framework in light of new data and system changes, at the same time acknowledging the required time for the EMR implementation to settle and mature.

## Results

Through the 3-stage process, we identified the key elements of the CBA framework, which will be presented below. It is noted that while these elements are partly guided by the literature (stage 1) and local practice (stage 2), their measurement and evaluation timeframe were carefully derived and guided by the economic value framework (stage 3). These elements can be adapted to fit the evaluation context (e.g., hospital and health systems of individual states or countries), healthcare delivery objectives and financing (e.g., high or low-middle income countries, or systems with universal healthcare versus privately funded healthcare), funding agencies and data availability (e.g., comprehensive and integrated versus rudimentary health information systems).

### Key CBA parameters

#### Counter-factual scenario

The counter-factual scenario here is a hospital *without* an EMR system operating in the same environment and time period as the hospital *with* an EMR. The counter-factual scenario is not the same as the hospital “before” the EMR system (or pre-EMR). Hospitals and health systems are dynamic; changes are continuously introduced, and therefore, a hospital “without” EMR might experience changes regardless of whether they are digitalised.

#### Timeframe

Following the ten-year vision of digital health reform developed by the local hospital and health authority, and confirmed during the stakeholder consultation phase, we selected a 10-year evaluation timeframe. The first six years capture the impacts enabled by digital transformation across Horizons 1 and 2, while the last four years captures emergent impacts of Horizon 3 (see Appendix).

#### Discount rate

In cost-benefit analyses, the discount rate is often used to reflect the preference for time, i.e., lighter weighting of future benefits and costs to current. The recommended rates for evaluation are 2%, 5% and 7% (Commonwealth Department of Health). It is noted that, however, discount rates should be selected to suit the country and context of evaluation.

#### Analysis perspectives

Four perspectives that represent relevant stakeholders of the EMR implementation were considered:

1. Market: the market CBA values all project inputs and outputs at their market prices (i.e. financial costs and revenues) and indicates whether the EMR investment is efficient from a market (or financial) perspective.

2. Private (the project owner): this perspective examines the EMR only from the individual hospital point of view, in which only their financial costs and revenues are used to calculate the net benefits (or profit). If the hospital pays taxes or receives subsidies for the EMR investment, these items will be included in the private analysis.

3. Efficiency: in the efficiency analysis, “economic prices” (also called shadow prices) are used for all relevant inputs and outputs, including those that have not been considered in the market CBA. The shadow price is defined as the true marginal cost or marginal benefit that reflects the opportunity cost of the resources and the economic value of the outputs. It can be the same or different from the market price. The efficiency CBA determines whether EMR investment is an economically efficient allocation of scarce resources.

4. Reference Group: the reference group is comprised of stakeholders deemed to be relevant to the decision-makers about the EMR investment. The CBA, from this perspective, shows the distribution of the project net benefits: who gains and who bears the cost of EMR. The reference group analysis is a feature that distinguishes this CBA framework [18] from a conventional CBA with a single bottom line (i.e. either net present value, internal rate of return, or profit).

The Referent Group include patients within the Hospital and Health Service catchment (P), staff of the hospital (S), the hospital as a legal entity (H), the Hospital and Health Service and Treasury as an entity (D). In contrast, the Non-Referent Group include the community outside the Referent Group (C - patients outside the catchment, the research community, and the primary care sector). This group is important in any health service investment; however, it is not directly relevant to the CBA in question.

**Fig. 3:**
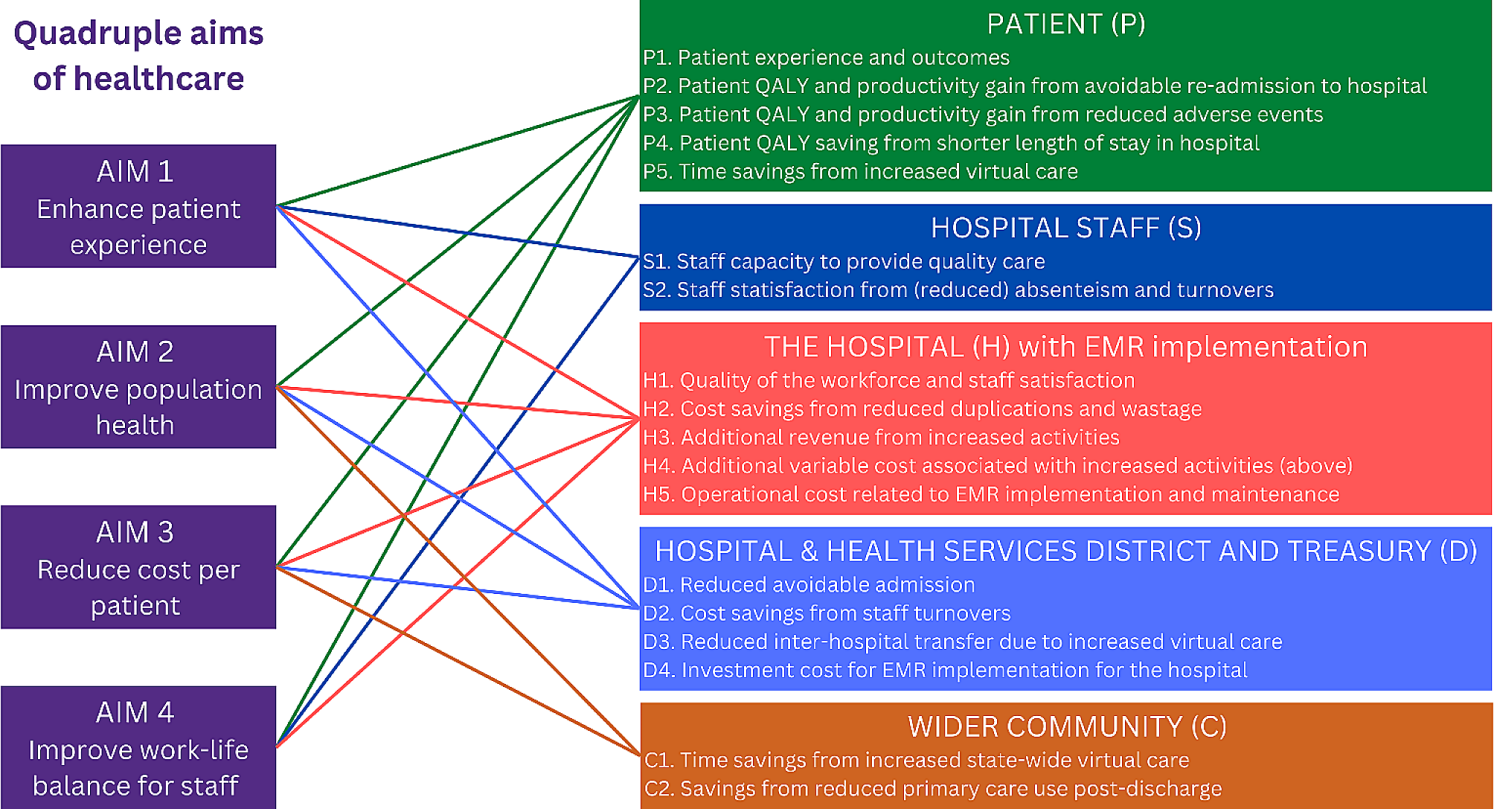
Mapping quadruple aims to cost and benefit items for the EMR implementation. *Abbreviation* QALY: quality adjusted life year; EMR: electronic medical records

The identified benefit and cost items are allocated to different stakeholders and mapped to the quadruple aim of health care (Fig. 3).

All benefits and costs included in the final CBA were those that met the two criteria: important and large (enough) impact. Insignificant impacts with small magnitudes, relative to other items do not add value to the analysis and therefore can be excluded. Extensive sensitivity analyses were conducted on all items included in the CBA, in both one-way and multi-way probability sensitivity analyses.

### Impacts of EMR implementation (benefits and costs)

In the framework, we identified 14 benefit and cost items (see Fig. 3). All items might require adaptation, addition or omission according to the evaluation settings. Three items (value of insurance and hope, research benefit from scientific spillovers, and value of improved Key Performance Indicators) did not have reliable data so we did not present their values but discussed their relevance to guide future data collection in the Appendix.

#### Patient benefit: improved experience (P1, P2, P3, P4)

The patient experience reflects both the health outcomes (objective measure) and quality of care experienced (patient-reported subjective measure) and is correlated with high-standard services. Patients are likely to have a better experience with the new model of care in digital hospitals, through two main channels: all their medical information is readily available for treating clinicians, leading to timely and more accurate treatment decisions (main channel), and to the patients themselves through self-monitoring via display dashboard (secondary channel). Better experiences follow the assurance for patients that clinicians have real-time and visible information for timely, precise care. In addition, patients would have more equal access to expert clinicians who are not in their neighbourhoods since EMR enables virtual care.

Patient experience is measured using standard PROMs and PREMs, and the value of patient experience can be elicited through the willingness to pay survey, completed by patients and their families. Complementary information for the value of patient experience can be derived indirectly from patients’ choice of treatment facility (e.g., digital hospital versus non-digital hospital providing the same care services).

The comparative improvement in patient experience, compared to the counter-factual scenario, is likely to diminish over time when EMR and EMR-related delivery models become the standard of care. Further, as variations in care quality reduce, thanks to improved precision and personalised care enabled by uniformed and comprehensive information, it will become increasingly difficult to generate further EMR-related quality and experience improvement.

#### Patient and health system benefit: reducing adverse events, both hospital-acquired and post-discharge (P2, D1, C2)

There are two types of post-hospital AEs: (i) serious AEs that require re-admission to the hospital and (ii) post-hospitalisation AEs that require medical attention by GPs. Both types of AEs can be greatly reduced by making better use of patient information through EMR implementation. As a result, both health system efficiency and patients’ health outcomes and quality of life will improve.

These benefits do not have a “market price” but they can be approximated by the willingness to pay for quality-of-life improvement and the opportunity cost of productive (or leisure) time loss incurred by the patients (for labour productivity). The savings from GP visits can be estimated using the predicted reduction of AEs that requires primary care services and the cost associated with providing GP service.

#### Patient benefit: shorter length of stay for patients (P4)

EMR implementation enables the capacity to deliver virtual care that is relevant during the Covid-19 pandemic [25] or similar situations in the future. In the traditional model of care (the counter-factual scenario), rehabilitation patients stayed for prolonged periods as clinicians monitor progress to safeguard against avoidable re-admission or AEs. With EMR-based virtual care, a proportion of patients could be discharged earlier to their local hospitals or receive earlier transition/community care with concurrent virtual care delivered by hospital staff. This new model would effectively reduce the length of stay and lead to quality-of-life gains since staying in a hospital is associated with poorer quality of life in patients who are not critically ill, compared to those who can access hospital-at-home.

#### Patient and health system benefit: time savings due to increased access and use of virtual care (P5, D3, C1)

Another envisioned benefit of digital hospitals is the increased use of virtual acute care. Compared to the counter-factual scenario, digital hospitals will deliver more virtual care. The increase is driven by two factors. First, the gradual rollout of digital technologies allows expert clinicians at one hospital to deliver virtual care to patients in other hospitals, especially those outside metropolitan areas. Second, both staff and patients at a digital hospital become increasingly familiar with virtual care delivery and are more willing to substitute it for physical care delivery. This rising acceptance has been shown clearly through the Covid-19 pandemic, where volumes of virtual care rose ten-fold in countries like Canada and New Zealand [26, 27], and between 50 and 90% of medical practitioners now offered virtual care in countries like Australia, France and some part of the US [28, 29]. This substitution effect happens for both outpatient and inpatient services, and for both patients within and outside of the hospital.

The increase in virtual care has impacts on patients, treating clinicians, the hospital and the health system overall. For patients, the benefits include reduced travelling time and financial expenses, increased access to expert clinicians without excessive wait time or being transferred from other facilities, and being discharged back to the community without compromising care continuity and quality. The opportunity cost reflects the value of time savings for patients, that is the value of other productive activities (including leisure time) that patients would be able to do should they not have to spend it on travelling to and/or staying in the hospital to receive care. The reduction in transfers leads to both time and financial savings for both patients (airfare, accommodation and accompanying carers) and the health system (ambulatory transfer services). For the hospital and health system, discharge earlier to the community releases beds for other patients, effectively reducing the waitlist or reserving the bed space for patients who need longer stay in the hospital (more complex cases). This leads to improvement in the efficient use of hospital resources and vertical equity (access and utilisation of hospital services by patients). Additionally, virtual care can provide specialist services to patients in regional and rural hospitals. This should result in fewer transfers from these areas to the metropolitan hospitals, leading to substantial savings that are not yet fully quantified elsewhere.

#### Staff benefit: improved productivity and capacity to provide quality patient care (S1, S2)

An indirect benefit of digital hospitals is \ improved productivity and workflow and the “value of knowing”. Digital hospitals are expected to increase the visibility of patient information and improve coordination (i.e., less time searching for needed information, including travelling time), which leads to improved productivity of labour. That is for the same amount of care to achieve the same health outcomes, it requires less time spent on unproductive activities. This shift can lead to improved staff, shorter lengths of stay and an increased number of patients.

The benefit for staff can be approximated by the perceived reduction in time spent on unproductive activities at work and the value of knowing the patients can be accessed anytime, anywhere (value of knowing). Unproductive activities include searching for information, checking to avoid medical errors, and waiting times for care coordination and medical results.

#### Hospital and health system benefits: medical input savings per patient (D2, H2)

The EMR implementation increases the standardisation of patient care, resulting in decreased wastage of health care inputs per patient. This is observed with small decreases in the total cost of radiology examinations, drug costs and laboratory testing because fewer duplicate or unnecessary inputs are ordered [8, 30]. Furthermore, a reduction will also occur in the stationery and paper-based costs following the transition to digital records. For all items, a reduction is expected on a per-patient basis; however, some categories will be considered as disbenefits because the increased patient load associated with EMR will result in increased costs for the activity. For the items – drug costs, radiology costs, and laboratory testing – an estimate could be made on the per-patient costs in the absence of EMR and separated into the categories of endoscopy, surgical, and rehabilitation patients. One might consider only inpatients for this analysis because outpatients might not require any of these inputs.

#### Hospital benefit: additional revenue (reimbursement) from increased activities (H3)

Increased services have implications on the cost, revenue and non-financial benefits. Another implied benefit of the EMR-induced productivity increase is a reduced waitlist for patients who otherwise do not have access to hospital services during the evaluation timeframe. This has several implications, including improved quality of life for many patients and additional use of medical resources that might be considered unnecessary.

#### Staff and health system benefits: high-quality workforce with EMR-based technologies (S1, H1)

Clinicians place a high value on their working environment, which consists of state-of-the-art medical technologies that enable healthcare innovations, a professional workforce, and collaborative opportunities to enhance their knowledge and experience and provide better care to patients. A digital hospital with EMR infrastructure is an example of this environment. It is therefore expected that digital hospitals would attract a workforce with higher-than-average expertise and experience for the same level of payment and be likely to have lower turnover and absenteeism rates, compared to its counterfactual.

For the first item – higher-than-average skilled workforce – we can use a two-scale difference in salaries to approximate the benefit for a digital hospital by having such a workforce. Note that part of this benefit inevitably translates into the higher productivity and quality of care captured by the patients. For the second item – lower turnover and absenteeism due to a “sticky” workforce with higher job satisfaction – the information can be sourced and adapted from the literature.

### Costs

#### Hospital and health system cost: investment (D4)

The investment covers the additional infrastructure expenditure compared to standard hospital commissioning. Examples include equipment, hardware integration and device uplift for end users. The investment value (or cost) is often straightforward because most items will be sourced from suppliers; hence, the financial values are available. Investment cost also factors in the reduction in investment costs compared to the traditional hospital setting (the counter-factual scenario). For example, if digital hospitals require less physical space for papers, printers, and storage, then this reduction should also be considered.

#### Hospital and health system cost: EMR-related operating cost (H4)

The standard operating costs specifically related to digital hospital implementation include EMR support services for staff and end-user device support and maintenance. Inputs for those cover labour (including indirect labour), software and applications, hosting services, and device replacement. Depending on the stage of EMR roll-out, other inputs might cover clinical resources to support rolled-out projects to accelerate adoption (immediate/short-term), and clinical enhancement and optimisation of the EMR to improve the clinical process (medium to longer-term). Note that these operating costs exclude the variable costs associated with healthcare delivery to patients, unless there is evidence that EMR implementation allows for service expansion.

#### Hospital and health system cost: additional healthcare related expenditure (H5)

Over the medium term, digital hospitals are expected to serve a higher volume of patients thanks to improved productivity of both staff and capital resources. Serving more patients requires additional variable costs associated with healthcare delivery. This cost can be estimated by the increased volume of services attributable to the EMR implementation, and the marginal variable cost per occasional of service. The difference in service volume, between the EMR scenario and the counter-factual, requires service projection from similar EMR implementation experiences, if any. Otherwise, a conservative assumption of volume change should be used. The marginal variable cost per occasional of service is sourced from hospital finance and/or the Hospital Independent Pricing Authority.

## Discussion

Digital transformation projects are expensive and take time to mature. Healthcare systems continue to plan large investments in digital technology and are struggling to justify funding that must be diverted from routine healthcare delivery. This paper presents the first framework for this purpose. The proposed eHealth-CBA framework is an augmented CBA that combines local knowledge and practice, healthcare delivery aims, methodological and empirical literature, and available data (inclusive of hospital-specific and those sourced from the literature).

The eHealth-CBA framework was developed in alignment with the quadruple aim of healthcare delivery. In previous studies that evaluated digital hospitals or EMR, none explicitly addressed all aspects of the quadruple aim. Cost per patient was the most important metric in those studies, followed by different metrics of population health, while patient experience, measured through the quality of life change, was considered in only one study [31]. Workforce experience was completely absent in the evaluation literature. Clinician burn-out is frequently observed in the hospital context and reported in the medical literature; yet it has not been quantified. Our framework accounts for impacts of digital transformation that have been long neglected, such as clinician experience and workflow, patient satisfaction and clinical outcomes both during and post-hospital admission. It explicitly itemizes the impact of EMR, both negative and positive benefits, then quantifies and values such impacts.

The alignment of the CBA with the quadruple aim also has another merit: avoiding overlapping and wastage within the catchment of health and hospital services. It has been shown in the literature that a systematic approach to project-cycle management (e.g., EMR investment) that is tightly linked to sector goals (healthcare delivery aims) can result in desirable outcomes in the medium and long-term (i.e., sustainability) [11, 31–34]. While the CBA result does not produce evidence of “improved efficiency” (due to reduced doubling-effort wastage), better accounting for benefit and cost items, and their true economic values imply that resources are used for the investments that have the highest returns.

This eHealth-CBA presents a range of information that informs the economic trade-offs between different stakeholders rather than presenting only one bottom line or number. It is based on the approach proposed by Campell and Brown [18] that allows for the comparison of scenarios from different perspectives: market (or financial) perspective versus efficiency (“true economic values”), and the distributions of net benefits across stakeholders. It zooms in the perspectives relevant to each value element, being costs or benefits. Together, the framework highlights who reap the benefits of the EMR investment and who bears the costs in each scenario, and how the trade-off between the stakeholders changes from one scenario to the next. Such information is essential for each individual hospital in the negotiation for EMR investment with the health and hospital authority and the budget holder (e.g., state or national financial department or treasury).

The framework aims to capture both financial and economic values of relevant and meaningful impacts of EMR implementation in hospitals and present them as distributions (of gains and loss) by relevant stakeholders. It explicitly displays the cost bearers and beneficiaries of such investment and focuses on the ultimate impacts: to patients, the hospital workforce, the health system, and society as a whole. This contrasts with the evaluation of EMR implementation as its own output: that is, “having an EMR per se” versus “the impacts of EMR implementation on relevant stakeholders”.

There is no doubt ascertaining values in some categories is difficult. However, if we do not explicitly value these categories, we allow implicit (often zero) valuation. Also, including such categories in the CBA framework will build awareness that these values do have a place in a more holistic CBA and spur enthusiasm for further efforts into capturing and refining these valuations. Additionally, developing the CBA framework can allow organisations to track the benefits of advancing digital maturity at a systems level and create a robust, evidence-based evaluation framework that can be transferable and repeatable and provide the opportunity to contribute to the science of structured digital maturity impact evaluations.

Several areas of potential uncertainty have been identified in the proposed framework. Firstly, the framework requires a rethink of the willingness of key decision-makers to pay thresholds. By increasing the number of elements of value, projects evaluated under a CBA framework will likely be recommended as desirable. Secondly, this approach relies upon the decision-makers to address the trade-off between equity and efficiency. Lastly, this is a CBA framework that carries the same robustness challenges that are well acknowledged in the evaluation literature. These include valuation issues and future uncertainty. Most CBA studies struggle with accurately valuing intangible or non-market goods like environmental quality or social well-being. Quantifying these factors into monetary terms can be subjective and contentious, leading to potentially biased assessments. As an appraisal and evaluation method, uncertainty is unavoidable. Predicting future costs and benefits with certainty is challenging. CBA requires making assumptions about future conditions and discount rates, which can significantly influence project evaluations. Uncertainty in forecasting economic, social, and environmental impacts can undermine the reliability of CBA outcomes.

Despite its early development stage in digital health and limitations, the application of CBA in this space can encourage a forward-thinking approach to digital hospital investments, promoting innovation, experimentation, and continuous improvement. By systematically evaluating the costs and benefits of emerging technologies and care delivery models, healthcare organizations can identify opportunities for innovation, anticipate potential risks, and adapt strategies to capitalize on new opportunities, ultimately driving transformative change in healthcare delivery. CBA also leverages data-driven approaches to evaluate the impacts of digital interventions in the era of big data and analytics, where digital hospitals generate vast amounts of clinical, operational, and financial data. When the healthcare industry is subjected to evolving regulatory requirements and policy initiatives, CBA provides a dynamic framework for evaluating the implications of regulatory changes, assessing compliance costs, and aligning digital strategies with broader policy objectives. By staying abreast of regulatory developments and conducting proactive CBA, healthcare organizations can navigate regulatory complexity, minimize compliance risks, and capitalize on emerging opportunities in the evolving healthcare ecosystem.

## Conclusion

Most funding decisions are made using financial models and net present values developed on cashable return on investments. Such approaches are not a good fit for the complex healthcare system with multiple important stakeholders and large investment uncertainty. Social cost-benefit analysis (CBA) is a valuable tool in appraisal and planning, and monitoring and evaluation of digital hospital investment, offering a multifaceted lens to evaluate the impacts, optimize investments, and navigate the complexities of digital transformation in healthcare. By harnessing the power of CBA, healthcare decision-makers can drive sustainable innovation, enhance patient outcomes, empower healthcare providers, and ensure the long-term viability and success of digital hospitals in the ever-evolving landscape of healthcare delivery. The augmented eHealth-CBA framework proposed in this original research project bridges the current chasm between financial business cases which fail to capture the non-cashable benefits of an EMR and the truly balanced evaluation of the complex impacts of an EMR. By placing the total economic value and quadruple aims of health care at the heart of our framework, we encourage future research efforts into those value elements such that they can be properly measured and valued.

### Electronic supplementary material

Below is the link to the electronic supplementary material.

Supplementary Material 1

## Data Availability

The paper describes a framework development. Therefore, no dataset is available.
