# Supplementary material for: Cashing in: cost-benefit analysis framework for digital hospitals
Source: BMC Health Serv Res. 2024 May 31;24:694. doi: 10.1186/s12913-024-11132-7 (PMC11143650; doi:10.1186/s12913-024-11132-7)
Supplement: Supplementary file 1 — Supplementary Material 1 [file 12913_2024_11132_MOESM1_ESM.docx]

Supplementary Electronic Material

# Three-stage development of the eHealth-CBA framework

## Stage 1 – Literature Review

A comprehensive scoping review to assess the current methodologies and proposed costs and benefits of EMR implementation published since 2000 (Figure SEM1).^1^ Three core themes were identified in the review, that was deemed important to consider when developing a digital health cost-benefit analysis framework:

- **Maturity of EMR and digital hospitals:** as EMR technologies mature, greater benefits have been observed. Most recent literature indicates that EMR has increasingly led to improvements in the quality of care and is becoming viable on a purely financial basis.
- **Economies of scope and scale:** current capabilities of EMR make it a solution for simple problems with the greatest impact in poor-performing healthcare systems. With further adoption, accruing positive network externalities enable EMR to solve more complex healthcare problems more effectively.
- **Alignment of EMR with health system goals:** implementation of the EMR was commonly undertaken with the primary outcome being the successful delivery of the EMR itself. This is short-term output-oriented, in contrast to medium and long-term outcome-oriented, in which ultimate outcomes such as improved population health, increased patient safety and quality of life, and improved satisfaction and well-being of the healthcare workforce. The latter is more aligned with the quadruple aims of healthcare, yet non-existence in the literature on digital hospital evaluation.

The literature search also pointed us toward approaches that could contribute to the framework development. Two approaches, the “elements of value” and the digital health benefit evaluation were deemed most complementary to the CBA approach.


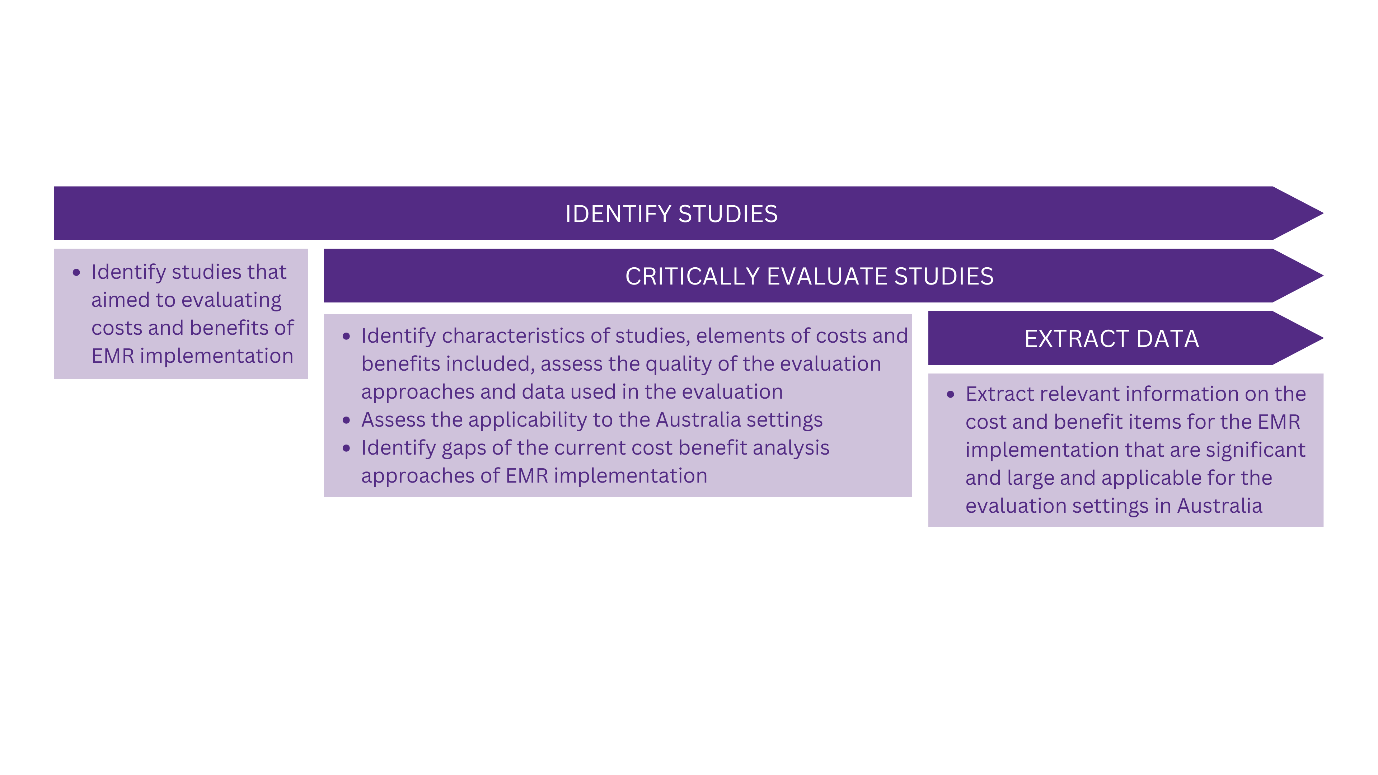


***Figure SEM1. Stage 1: Scoping review process.***

Abbreviation: EMR: electronic medical record

### The elements of value framework:

the ISPOR special task force report by Lakdawalla et al (2018)^2^ discusses a series of elements that warrant consideration in value assessments of health and medical technologies. They aimed to broaden the view of what constitutes value in health care and urged for new research of the additional elements.

The **light purple elements** in Figure SEM2 represent the current approach used to evaluate the impacts of health technologies and EMR in Australia and internationally. These include net costs - capturing the investment and operating costs as well as any cost savings realised by EMR implementation, the value of quality-adjusted life years (QALYs) – the standard economic measure capturing the impact of health technologies or interventions on extending the length of life, or quality of life.

The **pink elements** represent emerging elements of value that have been either discussed or incorporated in the economic evaluations, yet not in a systematic way. These include the value of labour productivity – capturing the value of the marginal product of labour by both patients and medical staff should there be evident that EMR has an impact on labour productivity, and the value of treatment adherence – reflecting benefits from improved effectiveness of medical treatments and health services because of higher standardisation and precision of EMR-enabled care models.

The **green elements** represent items of economic value that are not currently considered in most evaluations, either due to a lack of economic methods to translate the medical evidence into value, or data deficits. The fact that these elements outnumber both the green and blue elements highlights the current limitation in the healthcare evaluation framework. On the other hand, explicit discussions of those elements open up opportunities for future research in methodologies such that those elements are better measured and their values are captured appropriately in healthcare project assessments.


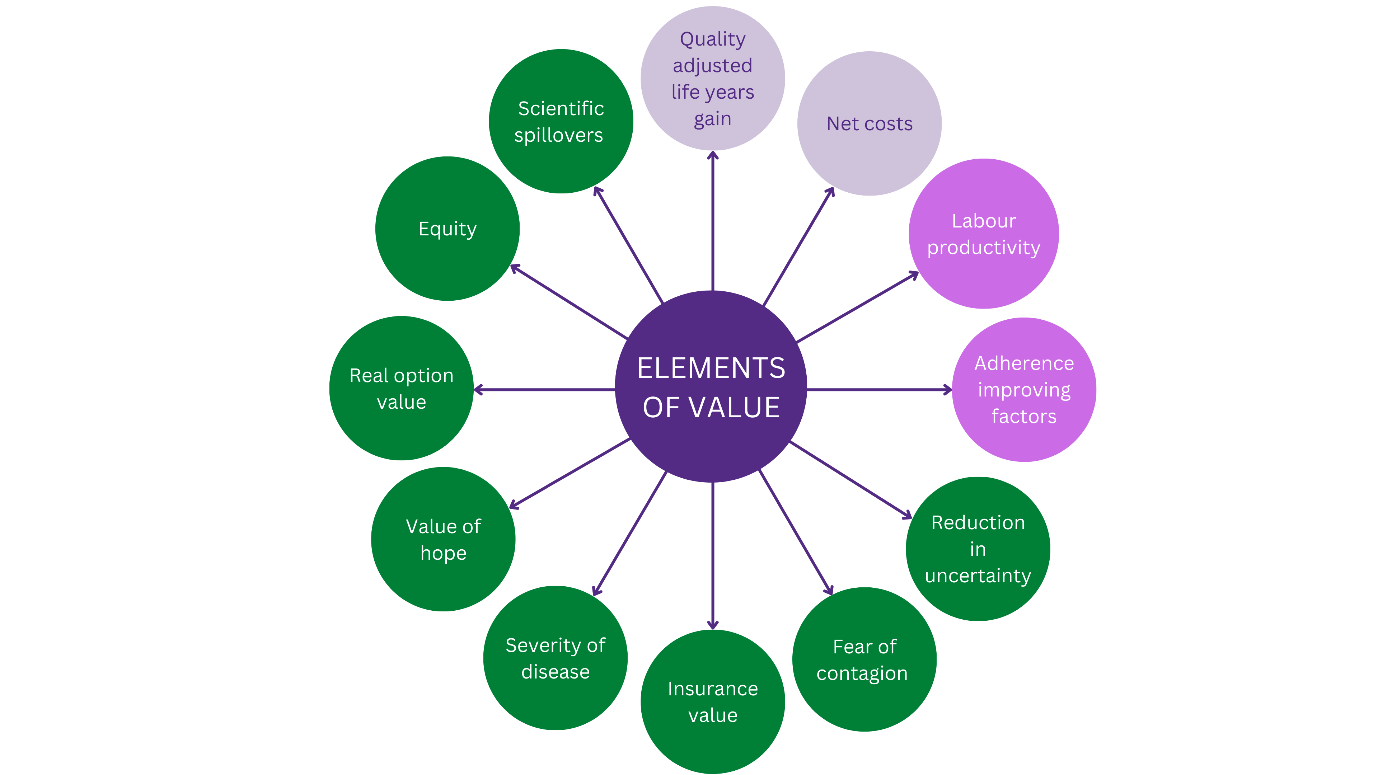


***Figure SEM2. Elements of value (adapted from Lakdawalla et al, 2018)***

### The ehealth benefit evaluation framework:

The framework was developed by Lau and Kuziemsky (2016),^3^ and presented elements of successful information systems in different settings,^4,5^ systematic reviews on the determinants of success in inpatient clinical information systems,^6^ and synthesis of results from health information system evaluations (Figure SEM3).^7^ It stops at naming the benefit items. The economic values of benefit items were not presented or discussed, making it impossible to use them for an economic evaluation.

The three main domains of net benefits include care quality, access (to care) and productivity: **“Care quality”** covers a wide range of categories and measures, including patient safety (e.g., preventable adverse events, near-misses and errors, reduction in patient risks and safety-related reportable adverse events, surveillance in the monitoring of specific populations for patterns), appropriateness and effectiveness (e.g., adherence and compliance with benchmark, policy or practice standards and guidelines, immunisation and testing and other relevant rates, continuity of care) and health outcomes (e.g., clinical outcomes, change in health status attributable to eHealth interventions). **“Access”** captures the ability of patients and providers to access services (e.g., availability, diversity and consolidation of eHealth-enabled services, timeliness, geographic, financial and cultural or linguistic, removal of inequitable barriers) and participation by patients and carers (e.g., patients’ self-management and access to their own information). **“Productivity”** includes efficiency (e.g., provider resource use, improvement in short-term outputs vs. inputs, and long-term in care continuity, improved health system management capability, improved patient efficiency and non-monetary effects), care coordination (e.g., care provided by the team, continuity of care across the continuum) and net cost (e.g., monetary avoidance and/or reductions, actual and projected savings).


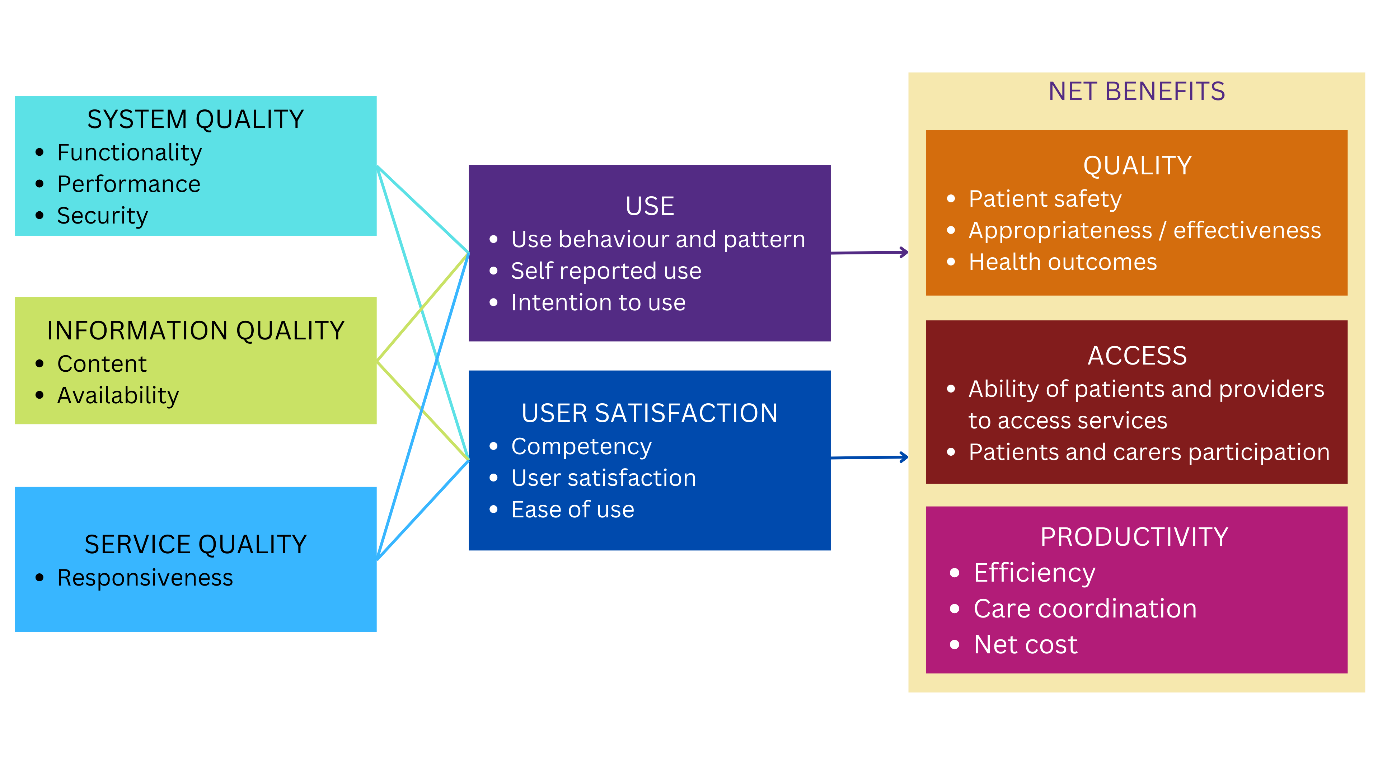


***Figure SEM3. eHealth Benefit Evaluation Framework, copyright 2016 by Canadian Health Infoway Inc. reproduced by Lau and Kuziemsky 2016***

## Stage 2 – Stakeholder consultation

A three-step systematic consultation process was adopted with a purposive sample of relevant healthcare stakeholders belonging to the Referent Group (Figure SEM4). Our medical academic centre is co-located on the hospital campus, and project members have existing research or operational relationships with healthcare stakeholders to invite their participation. For the digital hospital CBA analysis, the Reference Groups include patients, staff, the hospital as an entity, and the broader health district the hospital serves. Stakeholders who agreed to participate represent four main groups: the local health service, the healthcare system, clinicians and digital health peak body. In each stakeholder consultation a scribe took minutes representing stakeholder perspectives and preferences. Content analysis of the minutes was conducted by the project members following each consultation to aggregate findings to incorporate into improvements to the eHealth-CBA framework. Stakeholder consultation occurred from February to May 2021.

First, a 2-hour hybrid (in-person and online) consultative workshop (n=1) was organised immediately after the initial findings from the literature review were available (Stage 1). The workshop objective was to (1) discuss the literature findings and their implications for the CBA framework development, and (2) to begin to explore the feasibility, acceptability, and appropriateness of using the CBA approach^8^ to evaluate the economic value of EMR implementation. The workshop was conducted with members from all four stakeholder groups (n=15). Second, follow-up consultations (group meetings n=2) were conducted with a subset of stakeholders from all four stakeholder groups (n=13). The objective of the consultations was to (1) further discuss the feasibility and acceptability of CBA in evaluating digital hospitals and (2) to capture stakeholders' perspectives as the project continued. This is essential to reflect the practical experience and concerns of practitioners who directly implement and are affected by the EMR implementation as inputs for the CBA framework. Third, result consultations (presentations to governance groups n=3; individual executive meetings n=2) were conducted with the local health service and healthcare system stakeholders only (n=1 to 13). The objective of the result consultations was to confirm the feasibility and acceptability of the framework amongst relevant local stakeholders. The draft eHealth-CBA framework was presented for high-level feedback on measuring and valuing costs and benefits. Comments and suggestions were further incorporated into the final framework, including the current data limitations, and proposed future plans for data acquisition and repeated evaluation.


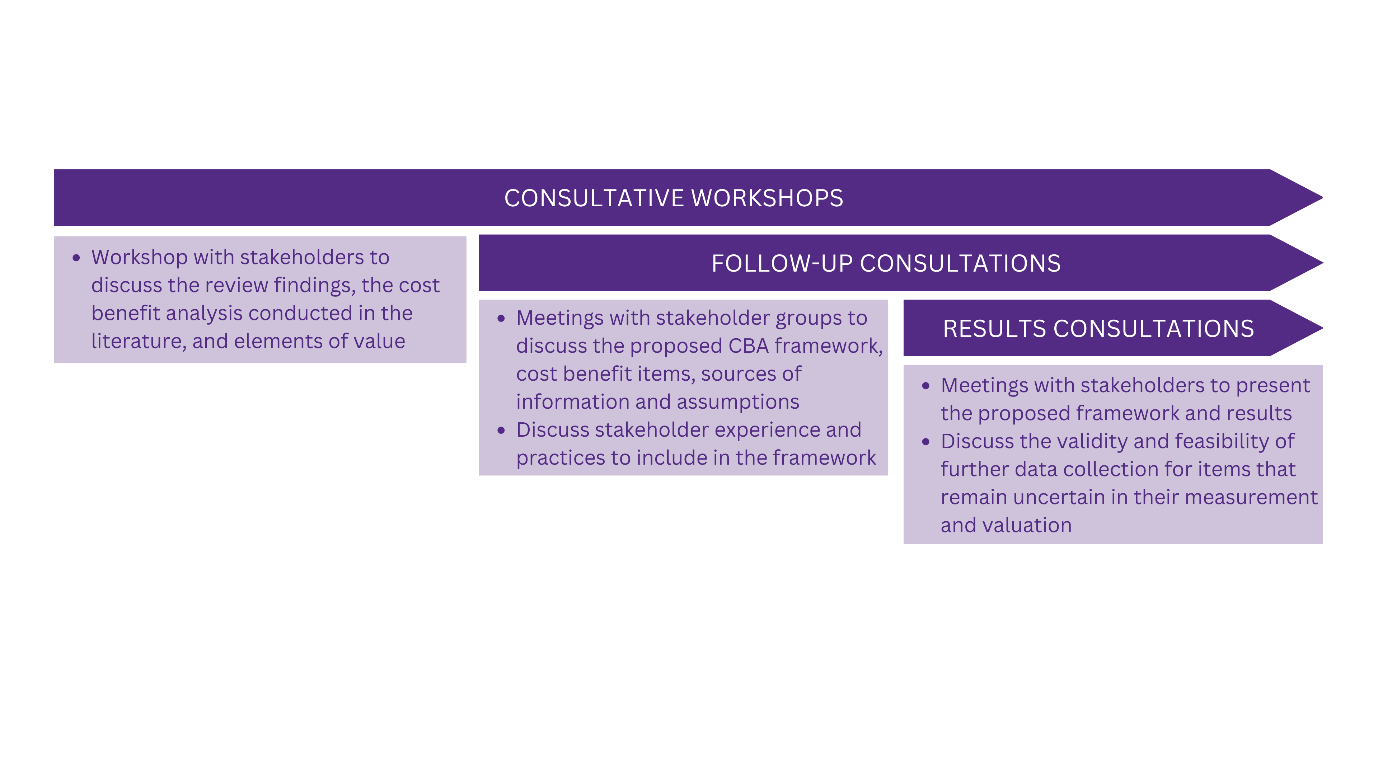


***Figure SEM 4. Stage 2: Stakeholder involvement, from initiation to finalisation of the CBA framework***

## Stage 3 – CBA framework development

This stage aims to develop a CBA framework to evaluate EMR implementation in hospitals, accounting for the quadruple aim of healthcare, the digital health transformation context of Australia, the existing literature on healthcare economics (evaluation methods, and “elements of value” framework), valuation of benefits and costs associated with eHealth implementation, and the local practice and experience by the stakeholder (hospital workforce, healthcare and hospital decision-maker and patients, consumers). The framework was improved through stakeholder engagement and feedback with a workshop and follow-up consultations (Stage 2) and aligned with the digital health horizon (Figure SEM5). This process has resulted in the first comprehensive economic evaluation framework developed for EMR implementation to inform decision-makers of the value of their digital investments (Figure SEM6).


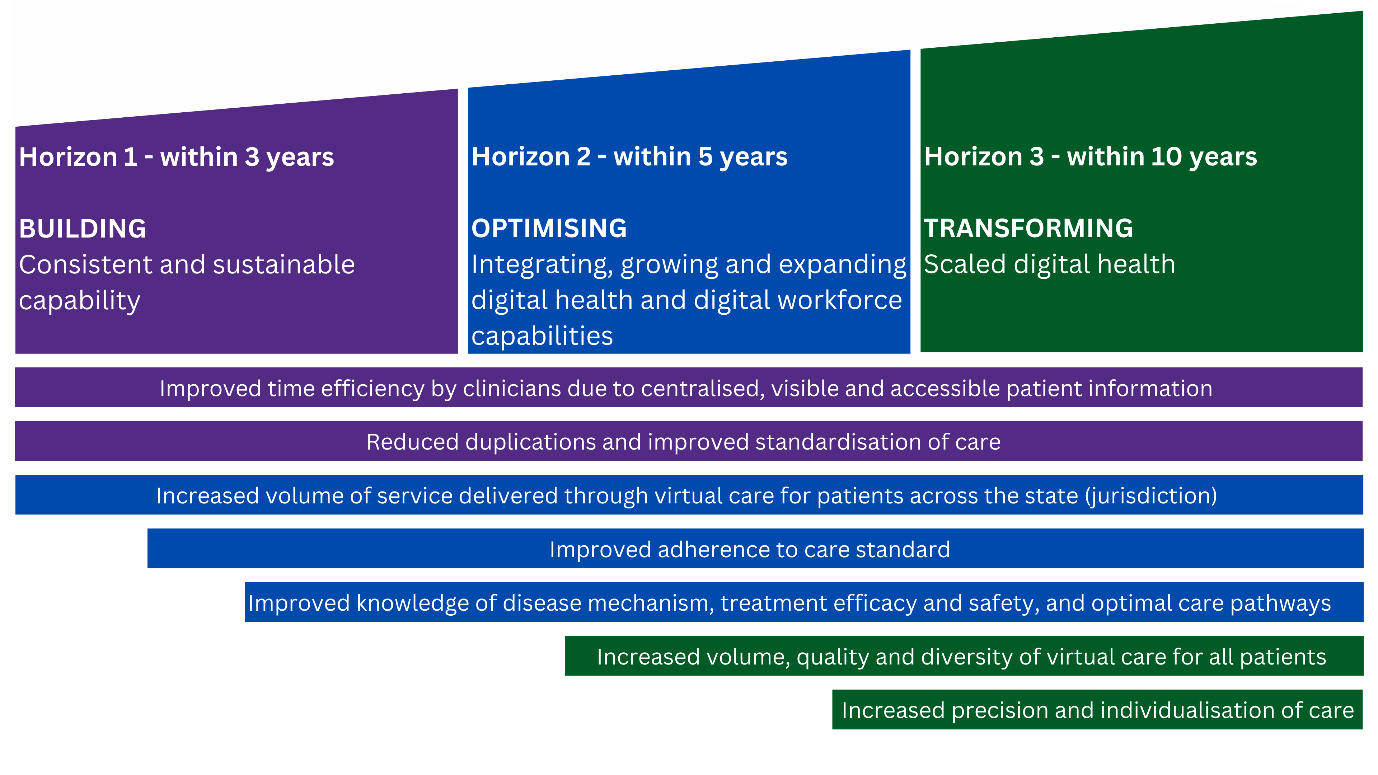


***Figure SEM5. Anticipated impacts of EMR implementation that leads to the identified costs and benefits***


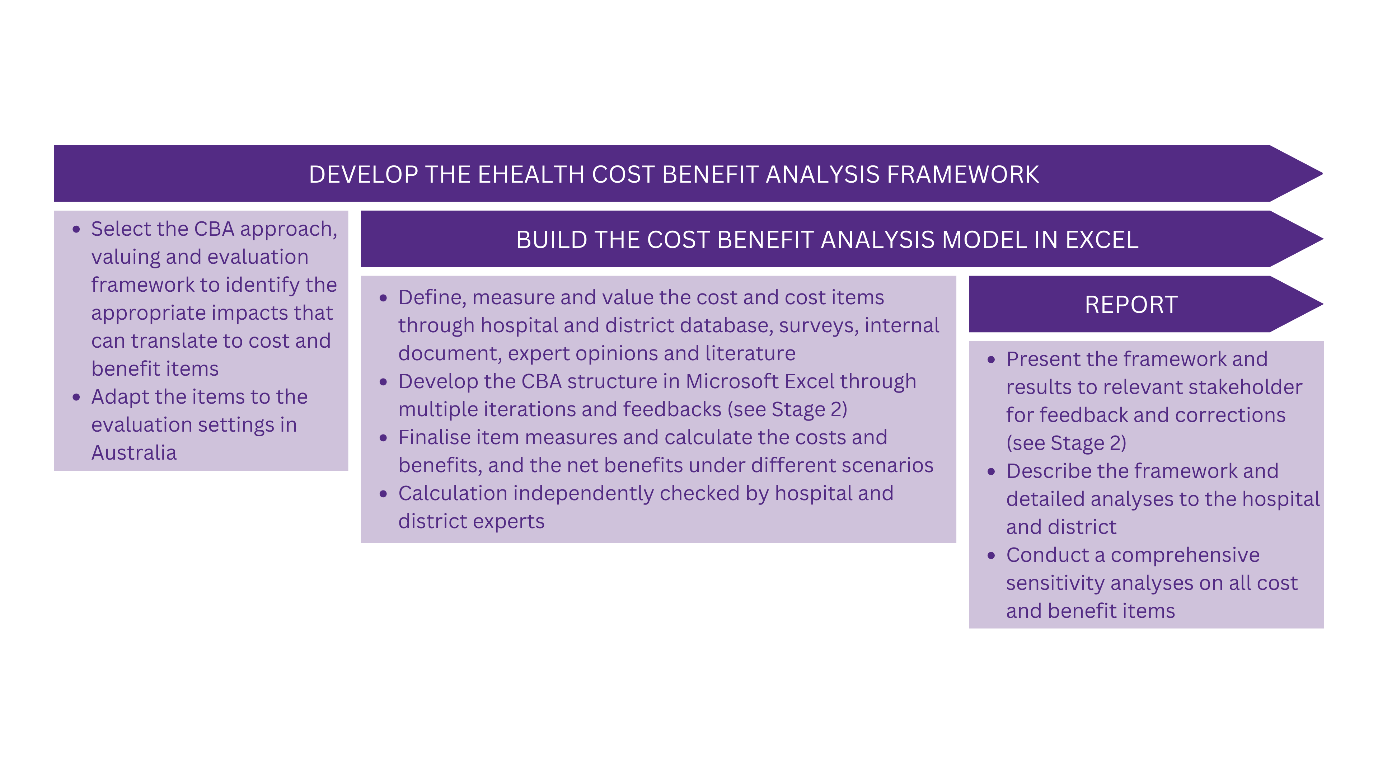


***Figure SEM6. Stage 3: eHealth CBA framework development and analysis***

# Additional benefit that are currently not qualified

### Patient benefit: value of insurance and hope

The value of insurance element reflects the psychological need for risk protection when falling ill. For healthy consumers, illness represents a risk, not a current condition. Hence, they have different perspectives on the value of advanced medical technologies. There is a “value in knowing” that new medical technology reduces the “physical risk” of getting sick, or the new services/treatments make the illness less unpleasant and thus improve well-being in the “sick” state. This extends not only to the value of knowing the effectiveness of technology, but also its availability when needed in the future, and the quality of the technologies or services provided. The EMR implementation is expected to increase the volume, quality and speed of access to patient and treatment data, leading to increased clinical knowledge and capacity for innovations in targeted models of care.

The value of hope is closely related to the value of reducing treatment uncertainty. It captures some individuals’ willingness to “gamble on a better outcome”, when there is high uncertainty around the treatment effect (both/either costs and/or outcomes). Health economists have long identified certain situations in which patients might become risk lovers who are willing to take gambles that do not focus solely on maximising expected length and quality of life. This reflects how some patients perceive the cost of uncertainty and the value of risk reduction, especially in the context where morbidity and mortality risk associated with the disease is high (high severity), and there are many unknowns about the technologies' effectiveness. For example, consider two different technologies that produce the same mean outcome, but one involves much greater uncertainty than the other. If patients value hope, they may gravitate to high-variance technology, hoping they will be one of the lucky few to benefit. In contrast, if patients are risk-averse, they may gravitate to the “safe bet” technology that insulates them against an unlucky outcome. Either way, they are choosing between two technologies that appear identical if we focus only on average costs and benefits.

### Society benefit: research benefits from scientific spillovers

This value element acknowledges the value of “positive externalities by future innovations”, and that certain health technologies might not have an immediate value (of treating a condition) but the knowledge about how the mechanism works might lead to other more valuable medical advancements in the future, even to treat very different diseases. The first technology might unlock new technologies or be a prerequisite for developing new drug technologies. Innovations with little stand-alone value still have merit if they stimulate follow-on innovation. To account for this benefit, one might consider including “scientific novelty” in the analysis, for example by using the number of research grants or quality improvement activities targeting service improvement as a proxy.

### Hospital and health system benefits: value of improved Key Performance Indicators

The ultimate outcomes of digital hospital services – like other healthcare services – are improved patient health outcomes and experience, population health, workforce quality and experience, and cost efficiency. Key Performance Indicators (KPIs) can provide specific measures of health services and systems and report the performance of all hospitals and health service districts. An improvement in KPI is a natural outcome of better quality and more efficient care services. KPI can be viewed as a signal of the commitment to providing quality healthcare; however, measuring the benefits of EMR on KPI might be challenging.

# References

1. Nguyen, K.-H. *et al.* Economic evaluation and analyses of hospital-based electronic medical records (EMRs): a scoping review of international literature. *Npj Digit. Med.* **5**, 29 (2022).

2. Lakdawalla, D. N. *et al.* Defining Elements of Value in Health Care—A Health Economics Approach: An ISPOR Special Task Force Report [3]. *Value Health* **21**, 131–139 (2018).

3. Lau, F. & Kuziemsky, C. *Handbook of eHealth Evaluation: An Evidence-based Approach*. (2016).

4. DeLone, W. H. & McLean, E. R. Information Systems Success: The Quest for the Dependent Variable. *Inf. Syst. Res.* **3**, 60–95 (1992).

5. Delone, W. H. & McLean, E. The DeLone and McLean Model of Information Systems Success: A Ten-Year Update. *J. Manag. Inf. Syst.* **19**, 9–30 (2003).

6. van der Meijden, M. J., Tange, H. J., Troost, J. & Hasman, A. Determinants of Success of Inpatient Clinical Information Systems: A Literature Review. *J. Am. Med. Inform. Assoc.* **10**, 235–243 (2003).

7. Lau, F., Kuziemsky, C., Price, M. & Gardner, J. A review on systematic reviews of health information system studies. *J. Am. Med. Inform. Assoc.* **17**, 637–645 (2010).

8. Campbell, H. F. & Brown, R. P. C. *Cost-Benefit Analysis : Financial And Economic Appraisal Using Spreadsheets*. (Taylor and Francis, 2015).
